# Evaluating agar-plating and dilution-to-extinction isolation methods for generating oak-associated microbial culture collections

**DOI:** 10.1093/ismeco/ycaf019

**Published:** 2025-02-11

**Authors:** Alejandra Ordonez, Usman Hussain, Marine C Cambon, Peter N Golyshin, Jim Downie, James E McDonald

**Affiliations:** School of Environmental and Natural Sciences, Bangor University, Bangor, LL57 2DG, United Kingdom; School of Biosciences, Institute of Microbiology and Infection, Birmingham Institute of Forest Research, University of Birmingham, Birmingham, B15 2TT, United Kingdom; School of Environmental and Natural Sciences, Bangor University, Bangor, LL57 2DG, United Kingdom; School of Biosciences, Institute of Microbiology and Infection, Birmingham Institute of Forest Research, University of Birmingham, Birmingham, B15 2TT, United Kingdom; School of Environmental and Natural Sciences, Bangor University, Bangor, LL57 2DG, United Kingdom; School of Environmental and Natural Sciences, Bangor University, Bangor, LL57 2DG, United Kingdom; School of Environmental and Natural Sciences, Bangor University, Bangor, LL57 2DG, United Kingdom; School of Biosciences, Institute of Microbiology and Infection, Birmingham Institute of Forest Research, University of Birmingham, Birmingham, B15 2TT, United Kingdom

**Keywords:** microbial isolation, oak microbiota, microbial culture collection, dilution-to-extinction, agar plating

## Abstract

Microbial isolation methods are crucial for producing comprehensive microbial culture collections that reflect the richness and diversity of natural microbiotas. Few studies have focused on isolation of plant-associated microbiota, with even less focus on forest trees. Here, we tested two isolation methods, (i) agar plating and (ii) dilution-to-extinction, for isolation of microbiota from leaf, stem, and root/rhizosphere tissues of oak trees. Microbial isolates obtained (culture-dependent) and the endogenous oak microbiota of the source tissue samples (culture-independent) were characterized by 16S rRNA gene and ITS community profiling. We found that the type of growth medium, incubation conditions, and sample type inoculated onto agar influenced the number of isolates and taxonomic richness of the isolates obtained. Most bacterial and fungal ASVs obtained from isolation-based approaches were only obtained using one of the two isolation methods, with only 12% of the ASVs detected in both. Moreover, the isolation methods captured microorganisms not detected by culture-independent analysis of the microbiota, suggesting these approaches can complement culture-independent analysis by enriching low-abundant taxa. Our results suggest that dilution-to-extinction and agar-plating approaches captured distinct fractions of the oak microbiota, and that a combination of both isolation methods was required to produce taxonomically richer microbial culture collections.

## Introduction

Metagenomics and other culture-independent approaches are widely used to characterize the composition and function of microbial communities in a broad range of ecosystems [1–3]. However, isolation and culturing of members of these microbial communities remains essential to better understand the functions and interactions within the microbiome and to dissect the individual roles, phenotypes, and physiological traits of its members [1, 2, 4].

Microbial isolation can complement findings revealed by culture-independent approaches. For instance, they can be used to detect rare or fastidious taxa underestimated in single-gene community profiling data [5], track microbial interactions in culture plates, and reconstitute complex microbial communities with specific compositions and metabolic capacities [1]. The latter approach constitutes a fundamental strategy for new research frontiers, such as microbiome engineering and synthetic microbial community design [6, 7]. In plant microbiome research, a variety of isolates obtained from plant-associated microbiotas have been used in experimental work to understand host-microbiome interactions [8, 9] and to demonstrate their efficacy for suppression of plant pathogens and promoting plant health [10–12].

However, the production of comprehensive microbial culture collections is hindered by a lack of rapid and efficient culturing methodologies [13]. This is especially true for tree species like oak, for which comprehensive isolate collections do not exist. Isolation methodologies face several challenges, including difficulties in environmental replication [14–16], metabolic interdependencies [14, 17], and selective conditions for rare taxa [18]. Collectively, these observations highlight the critical need to empirically validate suitable strategies to capture greater richness of microbiomes through optimizing *in vitro* growth conditions for large-scale isolation studies [13, 16, 19].

Agar plating using different growth media is a widely employed approach to isolate a broad range of microorganisms, including generalists and those with slow growth rates and specific growth requirements [12–14, 20, 21]. Standard culturing on agar plates can be efficient for capturing previously uncultured bacteria from complex microbiomes [22] and allows the documentation of colony morphology traits that can inform selective colony picking; however, it can be laborious, time-consuming, and often results in high redundancy of cultivated strains [1, 16].

Dilution-to-extinction overcomes some of the disadvantages of agar-based culturing and increases the throughput of isolation. Here, cell suspensions from plant tissues are serially diluted in liquid growth medium and dispensed into microculture plates. At the optimal dilution factor, theoretically, 30%–50% of wells in the microplate will result in the growth of a single isolate [23]. This method enables the separation of fast- and slow-growing strains in different wells which increases the likelihood of capturing low-abundant or rare members of the microbiota [13] and prevents the repeated isolation of fast-growing microorganisms. Nonetheless, it can still result in repeated isolation of dominant strains [1].

Agar plating and dilution-to-extinction have been used in combination to isolate a collection of 7000 microbial strains from the rhizosphere and phyllosphere of *Arabidopsis thaliana* plants [20]. Yet, these isolates originate from microbiomes of model plants or plants with agricultural importance, while microbiota from plant hosts with ecological, economic, and cultural importance, such as oak trees [24], have been explored to a much lesser extent. Tree-associated microbiota largely mediate tree responses to environmental challenges that threaten tree and forest health [25]. While much of the microbiome research on oak trees has been based on culture-independent methodologies [26–32], the early key insights to understanding changes in the oak microbiome under health and disease conditions were initially uncovered by microbial isolation studies [33].

Considering the requirement for representative culture collections of plant-associated microbiota and the need for empirical evidence to inform the selection of practical and effective isolation procedures, here, we tested the efficacy of different isolation techniques to cultivate members of the oak microbiota from oak leaf, stem, and root/rhizosphere tissue samples. The efficacy of agar plating and dilution-to-extinction was assessed in terms of number of bacterial and fungal isolates and taxonomic richness. This study constitutes a valuable tool to guide decisions on strategies to isolate the microbiota of plant hosts and produce plant microbial culture collections that act as reservoirs of strains with diverse applications in microbiome and plant health research.

## Materials and methods

### Sample collection and preparation of cell suspensions from oak tissue

**Figure 1:**
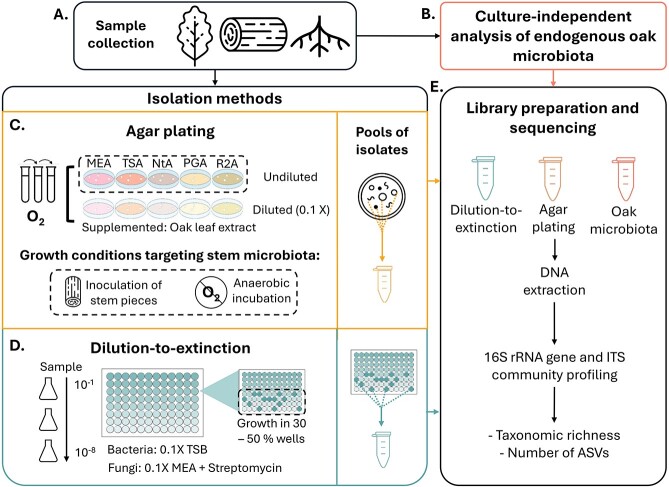
Methods tested for isolation of oak microbiota. (A) Leaf, stem and root/rhizosphere samples were collected from an oak tree and separated in two parts for 16S rRNA gene and ITS single gene community profiling to characterize bacterial and fungal communities, respectively. (B) A culture-independent analysis was conducted by extracting microbial DNA directly from the oak tissue. The second part of the samples was used for microbial isolation. (C) Agar plating isolations: all samples were serially diluted in 1X PBS and plated onto five diluted and undiluted growth media, and supplemented and non-supplemented agar plates and incubated aerobically. Additional growth conditions targeting stem microbiota were also tested. These included inoculation of stem pieces directly onto agar plates and anaerobic incubation. Dashed black lines correspond to growth conditions aimed at isolating stem microbiota. Colonies grown in each agar plate were pooled in a single sample. (D) Isolation by dilution-to-extinction: Cell suspensions were diluted in 0.1X TSB for bacterial growth and 200 μL was dispensed on wells of microculture plates. For fungal growth, serial dilutions in 1X PBS were dispensed on microwells containing MEA supplemented with streptomycin. Microwells exhibiting microbial growth were pooled in a single sample for amplicon sequencing. (E) Sequencing libraries were prepared from pools of isolates and from microbial DNA directly extracted from oak tissue for culture-independent analysis. Abbreviations: MEA: Malt extract agar, TSA: Tryptone soy agar, NtA: Nutrient agar, PGA: Potato glucose agar, R2A: Reasoner’s 2 agar, TSB: Tryptone soy broth.

Samples of leaf, stem, and root/rhizosphere (fine roots plus soil firmly attached) were collected from a 15-year-old oak tree (*Quercus robur*) located at the Henfaes Research Centre, Abergwyngregyn (Wales), and stored at 4°C until sample processing on the same day of collection (Fig. 1A). Samples were split into two parts, one was used for 16S ribosomal ribonucleic acid (rRNA) gene and internal transcribed spacer (ITS) profiling of the bacterial/archaeal and fungal communities, respectively, of the endogenous oak microbiota (culture-independent analysis, Fig. 1B) and the second part was used for microbial isolation (Fig. 1C and D). In all cases, samples were handled using tweezers and scalpel blades previously disinfected with 1% sodium hypochlorite for 20 min. Samples for culture-independent analysis were stored at −80°C.

Four groups of 8–10 mature oak leaves were collected from the low branches of the north, south, east, and west aspects of the oak tree and 12 leaves were randomly subsampled. Leaves were washed using sterile 1X phosphate-buffer sulfate (PBS, Sigma Aldrich, UK) to remove superficial dirt and dust and air-dried for 20 min in a laminar flow hood. Each leaf was split horizontally and vertically into four equal parts. Approximately 2 g of the remaining parts was ground in 10 ml of sterile 1X PBS to prepare a leaf cell suspension. A 1.5-cm-arch punch (C. S. Osborne, New Jersey, USA), previously disinfected by soaking in 70% ethanol for 20 min, was used to obtain a stem core (xylem, cambium, phloem, and outer bark) from the oak tree at breast height (1.3 m). The stem core was surface sterilized by three cycles of immersion of the sample in 1% sodium hypochlorite for 1 min followed by sterile 1X PBS for 1 min, and air-dried for 30 min in a laminar flow hood [34]. This step prevented the growth of microorganisms from the outside environment that could have contaminated the inner bark when the arch punch was inserted to collect the stem core. Two-thirds of the sample (350 mg) were sliced into 3 mm × 3 mm pieces [33]. Half of the stem pieces were inoculated directly on agar plates and the remaining half was used to prepare a stem cell suspension by grinding the sample using a mortar and pestle with 5 ml of sterile 1X PBS. Root/rhizosphere samples were collected using a spade, digging at a 20 cm depth along the largest root of the oak tree and 80 cm away from its base. Secondary oak roots (root tips with brown to yellow-brown coloration) were cut from the main oak root using a disinfected chisel and pruning shears. To prepare a rhizosphere cell suspension, roots, and the soil tightly attached to them (~2 g) were initially shaken in 40 ml of sterile 1X PBS for 10 min at 180 rpm [13]. Thicker root tissue in the sample (>3 mm diameter) was separated and ground in 10 ml of sterile 1X PBS using a mortar and pestle. Finally, both root and rhizosphere fractions were combined.

### Isolation of oak-associated bacteria and fungi

Briefly, two main isolation approaches were tested in this study, the first one was agar plating using varying media types, incubation conditions, and types of samples inoculated (Fig. 1C), and the second was dilution-to-extinction (Fig. 1D). The total number of bacterial and fungal colonies obtained on agar plates was recorded and this number represented the number of isolates recovered from this isolation method. Colony counts were not possible in the dilution-to-extinction approach, since liquid medium was used for bacteria, and fungal colonies on solid medium are not distinguishable in small surfaces such as a well of a 96-well plate; therefore, the number of wells with bacterial and fungal growth was used as a proxy for the number of isolates obtained in this method. Bacterial growth in wells was identified by increased turbidity compared with sterile medium, and fungal growth was distinguished by observing mycelial growth or yeast-like colonies on solid medium.

#### Microbial isolation by agar plating

Serial dilutions up to 10^−2^ and 10^−4^ were prepared from 1 ml of leaf and root/rhizosphere cell suspensions, respectively, in a total volume of 10 ml per dilution. A 100 μL-aliquot of each root/rhizosphere and leaf dilution and 100 μL-aliquot of undiluted stem cell suspension were spread in two replicates of five culture media: Reasoner’s 2 agar (R2A; Oxoid, Thermo Fisher Scientific, UK), nutrient agar (NtA; Oxoid), potato-dextrose agar (PDA; Oxoid), malt extract agar (MEA; Oxoid), and tryptone-soy agar (TSA; Oxoid) [20, 33, 35, 36]. We also tested diluted (0.1X) TSA and MEA medium, keeping the agar contents at a 2% concentration to allow full solidification. Both diluted and undiluted media were supplemented with oak leaf extract, prepared by grinding 1 g of oak leaves in 5 ml of 1X PBS using a mortar and pestle, centrifuging the suspension at 4000 rpm for 5 min, and filtering the supernatant using a 0.22 μm membrane. Sterile oak leaf extract was supplemented in the undiluted and diluted TSA and MEA media at a 2% v/v concentration (2 ml of oak leaf extract per 100 ml of medium). All plates were incubated under aerobic conditions for one week. In order to target stem microbiota specifically, we tested some additional growth conditions. Undiluted stem cell suspensions and stem pieces were inoculated directly unto the same five growth media mentioned above, and additionally in fastidious anaerobe agar (FAA). FAA was prepared by supplementing fastidious anaerobe broth (Neogen, Michigan, USA) with 2% bacteriological agar. The volume of stem cell suspension inoculated per plate was 100 μL and three stem pieces per plate were inoculated. Two sets of plates were prepared, the first one was incubated under aerobic conditions and the second set was incubated under anaerobic conditions using gas packs and anaerobic jars (Oxoid). The anaerobic atmosphere was monitored through color changes of the redox indicator resazurin (0.001 g/L) contained in FAA. In all instances, the total number of bacterial and fungal isolates obtained on each plate was recorded after 2, 5, and 7 days of incubation, except for plates incubated anaerobically, in which colony counts were conducted at day 5 and 7 to minimize disruption of the anaerobic environment in the jar.

#### Microbial isolation by dilution-to-extinction

To determine the optimal dilution factor for our samples (dilution at which 30%–50% of wells inoculated show microbial growth), 1 ml of the leaf, root/rhizosphere, and stem cell suspensions prepared as described above for agar plating, was also serially diluted up to 10^−8^ using 0.1X diluted tryptone-soy broth (TSB) and inoculated in microculture plates as follows: for bacterial growth, 200 μL of each dilution was dispensed in 24 wells of a microculture 96-well plate [13]; and for fungal growth, 10 μL of each dilution were inoculated on solid medium in another set of 24 microculture wells containing 200 μL of 0.1X diluted MEA supplemented with streptomycin to a final concentration of 50 mg/ml [37]. In total, 96-well plates were sealed with parafilm previously sterilized with UV light for 30 min, and incubated at 25°C under aerobic and static conditions for one week.

### Comparison of endogenous microbiota from oak tissue samples and oak microbial isolates

Bacterial and fungal isolates and endogenous bacterial and fungal microbiota from oak tissue samples were characterized by single-gene community profiling using the 16S rRNA gene and ITS region, respectively. The number of Amplicon Sequence Variants (ASVs) and the taxonomic composition obtained in both approaches was compared to determine how much of the oak microbial taxonomic richness could be cultured (Fig. 1E).

#### Extraction of microbial deoxyribonucleic acid from oak tissue samples

Deoxyribonucleic acid (DNA) extraction from oak leaf, stem, and root/rhizosphere samples were conducted following the phenol-chloroform method described by Griffiths et al. [38] with some specific modifications: Root/rhizosphere samples were initially flash-frozen in liquid nitrogen and ground using mortar and pestle, then mechanical lysis was conducted by bead-beating using 3 mm steel ball bearings for 1 min at 2.5 m/s in a PowerLyzer 24 Homogenizer (MoBio, UK). Samples were centrifuged at room temperature and treated with 4 μL of RNase A (Qiagen, Netherlands) 100 U/ml at 37°C for 30 min. Leaf and stem samples were flash-frozen in screw cap tubes by immersion in liquid nitrogen and then bead-beaten under the same conditions. All DNA samples were eluted in 50 μL of nuclease-free water and purified using the Monarch® Purification Kit (New England Biolabs, UK) following the manufacturer’s instructions.

#### DNA extraction from pools of oak microbial isolates obtained by agar plating

Bacterial and fungal colonies grown on agar plates were scraped off using sterile L-shaped spreaders and pooled in 1–5 ml of 1X PBS [39] (Supplementary Table 1). Colonies on agar plates were pooled per tissue type (leaf, stem and root/rhizosphere), and per agar type (R2A, NtA, PDA, TSA, and MEA). Additionally, colonies obtained under growth conditions targeting stem microbiota were pooled per incubation condition (aerobic and anaerobic) and per type of sample inoculated (stem cell suspension and stem pieces). Isolation negative controls were obtained by scraping zones of agar plates that were inoculated, but where no bacterial nor fungal colonies were observed. Bacterial and fungal cells were then pelleted by centrifuging at 4000 × g for 10 min and resuspended in 5% CTAB/phosphate (120 mM pH 8.0). DNA extraction was performed following the phenol-chloroform method described by Griffiths et al. [38] with the same modifications mentioned above.

#### DNA extraction from pools of oak microbial isolates obtained by dilution-to-extinction

Pools of bacterial isolates obtained by dilution-to-extinction in liquid medium were obtained by combining 20 μL of each well in a single tube. Fungal colonies grown on solid medium in 96-well plates were pooled by adding 20 μL of sterile 1X PBS in each well on top of the solid medium, pipetting to detach fungal cells from the agar and combining them into a single tube. Inoculated wells with no microbial growth were also pooled and sequenced as negative isolation controls. Microbial isolates were pooled per replicate and tissue type. Dilutions at which 30%–50% of the wells showed microbial growth were pooled separately from all other dilutions. Cells were pelleted and DNA extraction was conducted as described above.

### Library preparation and single gene community profiling analysis of the endogenous microbial community in oak tissue samples

For culture-independent sequencing analysis, polymerase chain reaction (PCR) amplification of the microbial DNA in oak tissue samples was performed using the universal primers 515F (5′-GTGBCAGCMGCCGCGGTAA-3′) and 806R (5′-GGACTACHVGGGTWTCTAA-3′) [40] to target the V4 region of the 16S rRNA gene for identification of bacteria and Archaea; and the primers ITS1 (5′-CTTGGTCATTTAGAGGAAGTAA-3′), and ITS2 (5′-GCTGCGTTCTTCATCGATGC-3′) [41] to target the ITS region for the identification of fungi. Both sets of primers contained a spacer and a tag for demultiplexing. DNA from the microbiota of oak tissue was amplified in 25 μL-reactions, containing 12.5 μL of 2X GoTaq Colorless Master Mix (Promega), a final concentration of 0.7 μM of each primer (either targeting V4 of 16S rRNA gene or ITS region), and up to 200 ng of DNA sample. To reduce the amplification of chloroplast and mitochondrial DNA from the plant tissue during 16S rRNA gene PCRs, chloroplast PNA clamp (5′-GGCTCAACCCTGGACAG-3′), and mitochondrial PNA clamp (5′-GTGAATTGGTTTCGAGA-3′) were added to the PCR reactions to a final concentration of 1 μM [42]. Cycling conditions were 95°C for 2 min, followed by 30 cycles of denaturation at 95°C for 45 s, primer annealing at 50°C for 60 s, PNA clamp annealing at 68°C for 10 s and extension at 72°C for 60 s, and a final extension step at 75°C for 5 min [42]. PCR amplification was conducted in three technical replicates per tissue type.

PCR products were quantified using the Quant-iT™ PicoGreen™ dsDNA Assay (Invitrogen) following the manufacturer’s instructions. 16S rRNA gene or ITS PCR fragments were then purified from any primer dimers or non-specific amplification fragments using AMPure XP reagent (Beckman Coulter, California, USA) at a bead/sample ratio of 0.9X, and eluted in 50 μL nuclease-free water. Purified PCR products were quantified in a Qubit fluorometer using the Qubit™ dsDNA HS Assay kit (Invitrogen, Thermo Fisher Scientific, UK). The final library was prepared by adding 65 ng of DNA per PCR product. Libraries were sequenced on the Illumina PE250 NovaSeq 6000 SP platform at NovoGene (Oxford, UK) to a depth of 40000 reads per sample.

### Library preparation and single gene community profiling analysis of oak microbial isolates

PCR amplification of DNA from oak microbial isolates obtained by agar plating and dilution-to-extinction was conducted using the primer sequences mentioned above, but these contained Illumina adapters, a spacer, and barcodes. Genomic DNA from microbial isolates was amplified in 25 μL-PCR reactions, containing 12.5 μL of 2X GoTaq Green Master Mix (Promega), 1 μL of each primer stock (10 μM) for a final concentration of 0.4 μM per primer, and up to 200 ng of DNA. Cycling conditions consisted of an initial denaturation step at 95°C for 2 min, followed by 30 cycles of denaturation at 95°C for 45 s, annealing at 50°C for 60 s and extension at 72°C for 60 s, and a final extension step at 75°C for 5 min [40].

2 μL of PCR amplicons were loaded onto a 1% agarose gel and visualized using SafeView DNA stain (NBS Biologicals Ltd, UK) in a Bio-Rad Gel Doc™ XR System (Bio-Rad Laboratories, Inc., California, USA). Aliquots of 10 μL of PCR product were combined in pools containing 10 – 15 samples that exhibited similar band intensities when visualized using gel electrophoresis. Pools of PCR amplification products underwent 1% agarose gel electrophoresis for 40 min at 100 V and were visualized in a Safe Imager™ 2.0 Blue Light Transilluminator (Invitrogen, California, USA). Bands corresponding to the expected fragment size for the 16S rRNA gene (~350 bp) and the ITS (~230) were excised from the gel using a scalpel blade treated with 10% bleach for 20 min and collected for purification with the QIAEXII Gel Extraction kit (Qiagen, Netherlands), according to the manufacturer’s instructions. Purified PCR products were quantified in a Qubit fluorometer using the Qubit™ dsDNA HS Assay kit (Invitrogen). The final library was prepared by combining 1–6 μL (10 ng) of PCR product to a final DNA concentration of 60 ng/μL. The final library was spiked with 2% PhiX DNA and loaded in an Illumina MiSeq Reagent Nano Kit v2 for cluster generation and sequencing by-synthesis (500 cycles, 250 bp paired-end) on the Illumina MiSeq platform (Illumina Inc., San Diego, CA, USA) at the Centre of Environmental Biotechnology (Bangor, Wales, UK).

### Bioinformatic analysis

Raw sequences were demultiplexed using Cutadapt v4.1 [43] specifying a maximum error rate of 15% and indel restriction. Sequences were processed using the ampliseq pipeline (v2.7.0 for bacterial and fungal isolations and bacterial culture-independent analysis and v2.11.0 for fungal culture-independent analysis) of the nf-core collection of workflows [44] utilizing reproducible software environments from the Bioconda [45] and Biocontainers [46] projects. The ampliseq pipeline was executed with Nextflow v23.10.0 [47]. Data quality was evaluated with FastQC [48] and summarized with MultiQC [49]. Primers were trimmed from the raw sequences with Cutadapt. Sequences were processed as one pool with DADA2 [50] to eliminate PhiX contamination, trim reads (to 200 bp forward and reverse), discard sequences shorter than 200 bp and sequences with >2 expected errors, correct errors, merge read pairs, and remove PCR chimeras. Between 0.1% and 91% 16S rRNA reads per sample (average 59%) were retained from samples derived from isolation, while between 77% and 86% reads per sample (average 83%) were retained from samples originated from culture-independent analysis. For ITS reads, between 0.1% and 99% reads per sample (average 38%) were retained from isolation samples and between 78% and 93% reads (average 88%) were retained from culture-independent analysis. Taxonomic classification was performed by DADA2 in the ampliseq pipeline, which uses the RDP Naive Bayesian Classifier algorithm [51] and the databases Silva 138.1 prokaryotic SSU [52] and UNITE v9.0 [52] for bacteria and fungi, respectively.

The output sequences from the nf-core/ampliseq pipeline (ASV tables, taxonomy tables, and associated metadata) were combined into phyloseq objects v1.44.0 [53] for downstream analysis in R v4.3.1 [54]. Datasets derived from culture-independent sequencing were curated by removing contaminant ASVs using the frequency method of the decontam package v1.24.0 [55]. ASVs classified as chloroplast and mitochondria were also removed (67285 reads removed, corresponding to 26% of the total initial data set). ASVs present in PCR negative controls were assessed as follows: If an ASV was present in the negative controls with a read count lower than in the sample, it was assumed that the ASV was not derived from external contamination and the number of reads of that specific ASV in the negative control was subtracted from the read count of the ASV in the sample. On the contrary, if the ASV had a higher read count in the negative control than in the sample, the ASV was removed from the sample. Finally, ASVs not assigned to phylum level and low abundant ASVs (read count per sample < 100 bp) were filtered from the data set.

For samples obtained from the microbial isolation, ASVs present in the DNA extraction and PCR negative controls with a read count higher than in the true samples were also filtered from the dataset. Additionally, ASVs not assigned to phylum level, low abundant ASVs (total read count per ASV < 100 bp), and ASVs assigned to non-bacterial taxa (i.e. chloroplast or mitochondria) were also removed. After all filters were applied to both data sets (isolation and culture-independent analysis), we obtained a data set comprising 171 861 16S rRNA reads and 267 724 ITS reads (Supplementary Table 2.).

Since the abundance of ASVs in microbial isolates do not necessarily mirror the ecological patterns of community composition in the microbiome, ASV read counts from isolation samples were transformed into binary data. Composition bar charts and heat maps were produced based on the number of ASVs per taxonomic level present in each sample. Venn diagrams of the number of ASVs that were common to different sample types – agar plating, dilution-to-extinction, and culture-independent analysis of the endogenous oak microbiota – were created using the ggVenn package v0.1.10 [56]. Unweighted Unifrac distances were calculated using the function “distance” from the phyloseq package v1.44.0 and heat taxa trees were created using the R package metacoder v0.3.7 [57]. All figures were produced using the package ggplot2 v3.4.2 [58].

For the statistical analysis, Kruskal–Wallis tests were applied to compare the number of isolates and ASVs between tissue types and isolation methods (agar plating and dilution-to-extinction) and between growth conditions (type and dilution of growth media, supplementation, incubation, and type of sample inoculated) using the R package stats v4.3.1 [54]. Jaccard distance matrices between communities obtained using different isolation approaches and sample types were calculated using the “distance” function of the phyloseq package. Permutational multivariate analysis of variance (PERMANOVA) tests were performed based on these distance matrices using the function “adonis2” from R package vegan v2.6-4 [59]. Comparisons between communities from different tissue types (leaf, stem and root/rhizosphere) and agar media (R2A, NtA, PGA, MEA, and TSA) and their interactions were tested with the formula “distance ~ medium ^*^ tissue” for agar plating, and the formula “distance ~ tissue” for dilution-to-extinction. Communities obtained from aerobic incubation and inoculation of cell suspensions were compared with those obtained from conditions targeting at stem microbiota (anaerobic incubation, inoculation of stem pieces) using the formula “distance ~ stem_growth_condition”. Finally, communities isolated by agar plating and dilution-to-extinction from each tissue type, and their interaction, were compared with the formula “distance ~ isolation_method ^*^ tissue”. All PERMANOVA tests were performed with 10 000 permutations. Significant differences between the number of ASVs per genus captured by agar plating and dilution-to-extinction were determined by Fisher's exact test from the R package stats.

## Results

### Varying growth conditions influence the number and taxonomic richness of microbial isolates obtained by agar plating

#### Taxonomic richness and composition of isolates from different tree tissues

**Figure 2:**
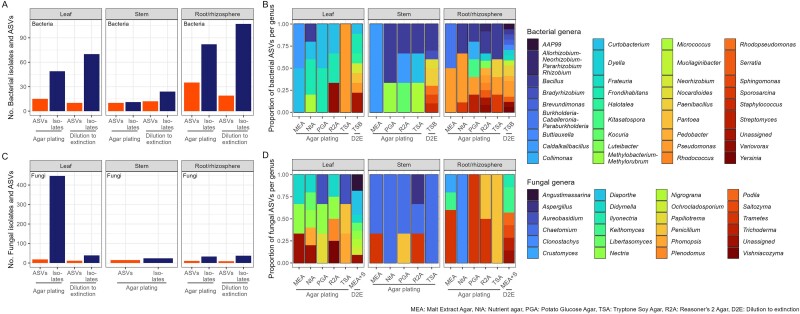
Bacterial and fungal members of the oak leaf, stem, and root/rhizosphere microbiota isolated by agar plating and dilution-to-extinction. (A) Comparison of the number of bacterial isolates and ASVs between isolation methods, (B) taxonomic composition of bacterial isolates obtained in five different growth media (agar plating) and using dilution-to-extinction, (C) comparison of the number of fungal isolates and ASVs between isolation methods, (D) taxonomic composition of fungal isolates obtained in five different agar media (agar plating) and using dilution-to-extinction. The number ASVs and the taxonomic compositions were obtained by characterizing pools of isolates using single-gene community profiling of the 16S rRNA gene, for bacterial isolates, and the ITS region, for fungal isolates. The number of ASVs per each bacterial and fungal genus was calculated and the proportion of ASVs per genus is displayed in stacked bar plots (B and D). No fungal growth was observed in the dilution-to-extinction method, hence, Fig. 2D only represents isolates obtained by agar plating. The growth media used were MEA, NtA, PGA, TSA, and R2A. Abbreviations: D2E: Dilution-to-extinction.

Oak leaf, stem, and root/rhizosphere cell suspensions were inoculated onto five different agar media. Subsequently, the number and taxonomic richness of isolates (16S rRNA gene and ITS profiling) was determined for each tissue type and growth medium. The number of bacterial and fungal isolates and ASVs obtained by agar plating varied significantly between types of tree tissue (Kruskal–Wallis *P*-value <.05, Supplementary Table 3, Fig. 2A and C). Root/rhizosphere samples yielded the highest number of bacterial isolates (189 isolates and 54 ASVs) followed by leaf (119 isolates and 25 ASVs) and stem (55 isolates and 34 ASVs). In contrast, fungal isolates were more numerous in leaf samples (486 and 30 ASVs), followed by root/rhizosphere and stem samples (70 isolates and 20 ASVs, and 49 isolates and 18 ASVs, respectively).

Based on 16S rRNA gene and ITS profiling of isolates, the composition of bacterial and fungal isolates also varied between the types of tree tissue (PERMANOVA *P*-value <.01, Supplementary Table 4, Fig. 2B and D, respectively). ASVs of the bacterial genera *Bacillus, Pseudomonas* and *Paenibacillus,* and the fungal genera *Trametes* and *Keithomyces* were dominant in the rhizosphere. The leaf bacterial isolates were mostly assigned to the genera *Frondihabitans* and *Curtobacterium* and the fungal isolates to the genera *Nectria* and *Didymella*. The stem bacterial and fungal isolates were classified into fewer genera, *Bacillus*, *Caldalkalibacillus*, and *Paenibacillus* and *Chaetomium* being the most abundant. Most of these genera have been previously reported as members of plant microbiomes [60–66].

#### The impact of growth medium on richness of isolates

The number of bacterial and fungal isolates and ASVs captured by agar plating was also variable between agar media, although these differences were not significant (Kruskal–Wallis *P*-value >.05, Supplementary Table 3). For bacteria, R2A yielded the highest number of isolates and ASVs (50 and 18 respectively). For fungi, the highest number of ASVs was achieved in MEA (11 ASVs and 100 isolates), although in PGA more isolates were obtained (*n* = 111), but representing only 7 ASVs, suggesting redundancy in the obtained isolates (Supplementary Fig. 1). We also identified 22 bacterial and fungal ASVs, belonging to different genera, that grew in only one specific medium (Supplementary Table 5). Fifteen of these ASVs (belonging to the bacterial genera *Kitasatospora* and *Streptomyces*, and the fungal genera *Crustomyces* and *Keithomyces*) were obtained from rhizosphere samples.

#### The impact of diluted and supplemented growth medium on number of isolates

**Figure 3:**
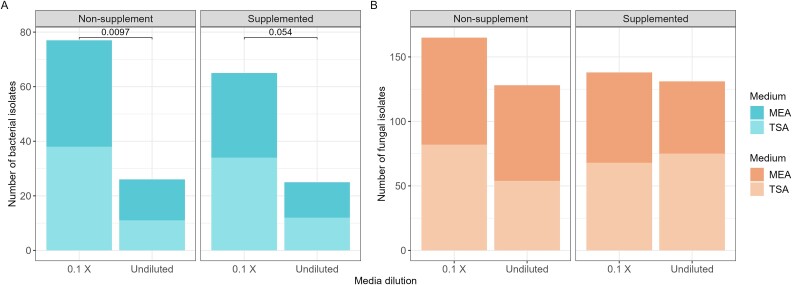
Isolation of bacterial and fungal members of the oak microbiota using non-supplemented and supplemented diluted and undiluted growth medium. (A) Number of bacterial isolates obtained from the oak root/rhizosphere, stem, and leaf microbiota, (B) number of fungal isolates obtained from the oak root/rhizosphere, stem, and leaf microbiota. The nutrients of the growth media TSA and MEA were diluted 10-fold while keeping a full-strength agar concentration of 2% for complete solidification. Diluted and undiluted agar media were supplemented with a final concentration of 2% of oak leaf extract. *P*-values were obtained by comparing the number of bacterial and fungal isolates in a nonparametric Kruskal–Wallis test.

We tested the impact of diluting the growth medium, or supplemenating the growth medium with oak leaf extract, on the number of bacterial and fungal isoaltes in leaf, stem, and root/rhizosphere. Overall, supplementation of agar with oak leaf extract did not result in an increased number of isolates compared with the non-supplemented medium. However, the use of diluted medium did yield a statistically higher number of bacterial isolates compared with undiluted medium (Kruskall–Wallis *P*-value <.05. Supplementary Table 6) (Fig. 3A and B).

#### Isolation of oak stem microbiota

**Figure 4:**
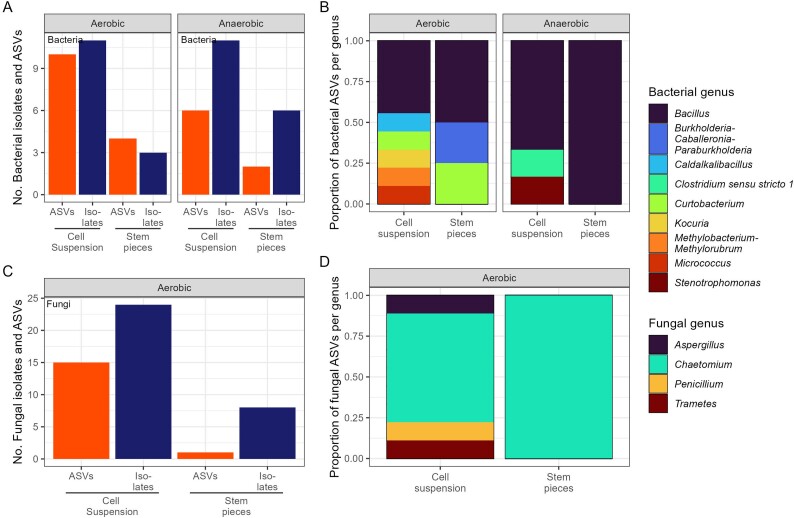
Oak bacterial and fungal isolates obtained by agar plating under growth conditions targeting stem microbiota. (A) Comparison of the number of bacterial isolates and ASVs obtained in a combination of five growth media (NtA, MEA, PGA, R2A, and TSA) under aerobic incubation and with inoculation of stem cell suspensions and stem pieces vs a combination of six growth media (addition of FAA agar) under anaerobic conditions, and with inoculation of stem cell suspensions and stem pieces, (B) taxonomic composition of bacterial isolates, (C) number of fungal isolates and ASVs obtained under aerobic conditions using the growth media and sample inoculations described above, (D) taxonomic composition of fungal isolates. Isolates obtained in a combination of five or six growth media were pooled and the number of ASVs and taxonomic composition of the isolates was determined by single-gene profiling of the 16S rRNA gene, for bacterial isolates, and the ITS, for fungal isolates. The number of ASVs per each bacterial and fungal genus was calculated and the proportion of ASVs per genus is displayed in stacked bar plots (C and D). The growth media used were MEA, NtA, PGA, TSA, R2A, and FAA.

The microbiota of tree stem tissue is one of the least explored microbial communities in the literature [67, 68]. Here, in addition to the conditions explained above (aerobic growth of cell suspensions on 5 medium types), we tested anaerobic isolation, an additional anaerobic growth medium (FAA), and isolations from stem tissue pieces inoculated onto agar plates. These additional isolation approaches yielded 20 bacterial strains (12 ASVs) (Fig. 4A), which represented 39% of the total number of bacterial isolates obtained from the stem using all approaches (31 bacterial isolates and 22 ASVs, Kruskal–Wallis test *P*-value >.05, Supplementary Table 7). This included five additional bacterial ASVs belonging to the genera *Bacillus*, *Burkholderia–Caballeronia–Paraburkholderia*, *Stenotrophomonas*, and *Clostridium*; the latter three genera were only isolated using these additional approaches (Fig. 4B). A small number of fungal isolates and ASVs were recovered using these additional growth conditions (Fig. 4C), however, no additional fungal genera were detected (Fig. 4D).

### Isolation by dilution-to-extinction.

Leaf, stem, and root/rhizosphere cell suspensions were serially diluted in 0.1X concentrated TSB medium and dispensed in 96-well plates to select optimal dilution factors where 30%–50% of the inoculated wells displayed microbial growth [13]. The optimal growth of bacteria and fungi from root/rhizosphere samples was achieved at dilution factors of 10^−5^ and 10^−2^, respectively, while in leaf samples, the optimal dilution factors were 10^−3^ for bacteria and 10^−2^ for fungi (Supplementary Table 8). In contrast, wells inoculated with stem suspensions only exhibited bacterial growth at 10^−1^ dilution, and no fungal growth was observed (Fig. 2). This is concordant with other reports of low number of isolates recovered from stem tissue compared to other plant compartments [68, 69].

To assess the richness of cultivated microorganisms obtained by dilution-to-extinction, biomass from all dilutions was pooled and characterized by single-gene community profiling. Similar to isolates obtained by agar plating, the identity of bacterial isolates captured by dilution-to-extinction differed significantly between tissue types (PERMANOVA *P*-value <.01. Supplementary Table 9). Despite testing a greater number of growth conditions using agar plating compared with dilution-to-extinction, the dilution-to-extinction approach captured more ASVs than any of the five agar media tested, (Fig. 2B and C), with a statistically significant difference between fungal ASVs (Kruskal–Wallis *P*-value <.05. Supplementary Table 10). This highlights the advantage of dilution-to-extinction in recovering greater richness of the natural microbiome. The most detected bacterial genera obtained by dilution-to-extinction included *Frondihabitans, Bacillus*, and *Pseudomonas* (Fig. 2B) and the fungal genera *Keithomyces* and *Diaporthe* (Fig. 2D).

### Agar plating and dilution-to-extinction capture different fractions of the cultivable oak microbiome

Across all tissue types, 162 bacterial isolates representing 40 ASVs were obtained by agar plating and 201 wells containing growth in dilution-to-extinction plates, representing 34 ASVs, were obtained by dilution-to-extinction (Fig. 2A). The bacterial ASVs obtained by both isolation methods were classified into 24 genera belonging to the phyla Proteobacteria (50%), Firmicutes (32%), Actinobacteriota (14%), and Bacteroidota (5%). Meanwhile, 26 fungal ASVs were obtained from 529 isolates cultured by agar plating, and 18 ASVs from 76 wells containing growth obtained by dilution-to-extinction (Fig. 2C). These were classified into 16 genera belonging to the phyla Ascomycota (76%), Basidiomycota (20.5%), and Mortierellomycota (2.6%).

**Figure 5:**
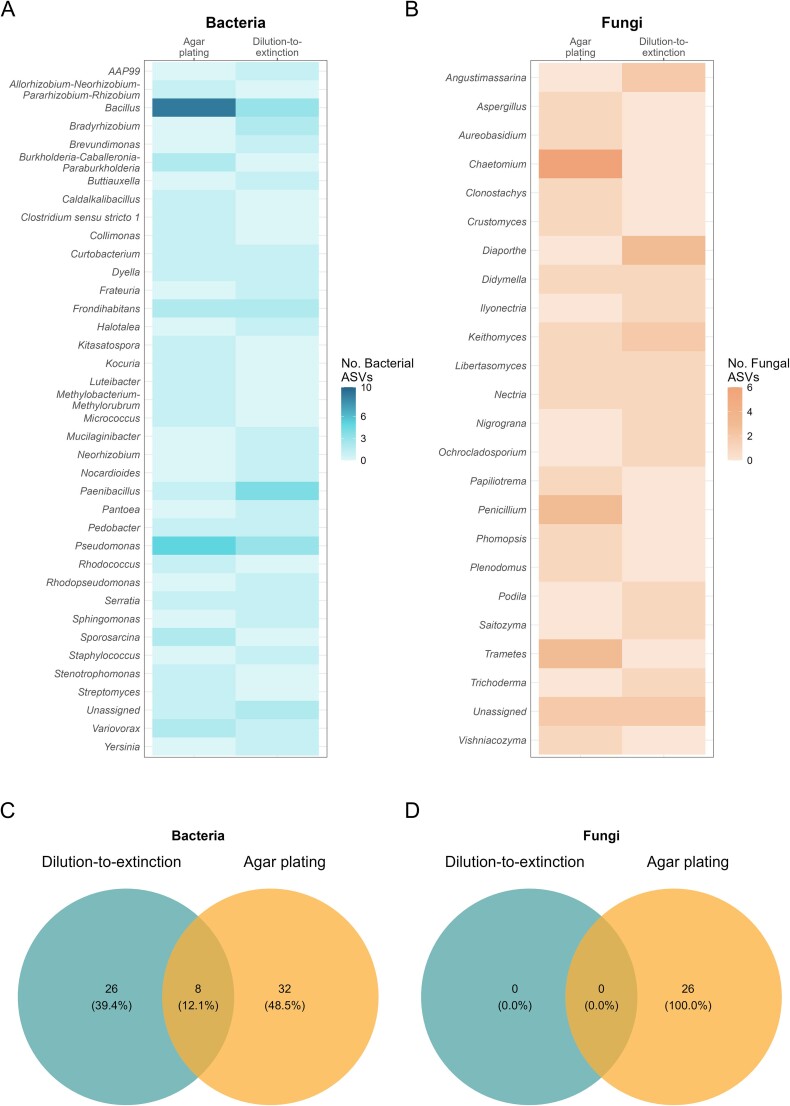
Comparison of taxonomic richness and composition of oak bacterial and fungal isolates obtained by agar plating and dilution-to-extinction. (A and B) Heat map of the number of ASVs per genus obtained from bacterial and fungal isolates of the oak microbiota isolated by agar plating and dilution-to-extinction, (C and D) number and percentage of bacterial and fungal ASVs that were captured uniquely by agar plate or dilution-to-extinction and simultaneously by both isolation methods. Microbial isolates were characterized by single-gene profiling using the 16S rRNA gene, for bacteria, and the ITS, for fungi.

Distinct bacterial and fungal ASVs were obtained by the agar plating and dilution-to-extinction isolation methods. These differences were significant for both bacterial and fungal isolations when comparing the ASVs obtained by each method per tissue type (PERMANOVA *P-*values ≤.05, Supplementary Table 11). For instance, ASVs assigned to the bacterial genera *Bacillus* and *Pseudomonas* were more commonly captured by agar plating, while *Paenibacillus* were more common in dilution-to-extinction (Fig. 5A). Similarly, ASVs belonging to the fungal genera *Chaetomium*, *Trametes*, and *Penicillium* were mostly dominant in agar plating, while *Diaporthe* was more enriched in dilution-to-extinction (Fig. 5B). These differences were only significant for the bacterial genus *Bacillus* and the fungal genus *Chaetomium* (Fisher’s exact test *P*-value <.05, Supplementary Table 12). Overall, 32 bacterial (49%) and 21 fungal (54%) ASVs were obtained exclusively through isolations by agar plating while 26 bacterial (39%) and 13 fungal (33%) ASVs were obtained only by dilution-to-extinction. Only 8 bacterial and 5 fungal ASVs were captured by both isolation methods. This corresponds to 12% of the total bacterial ASVs and 13% of the total fungal ASVs obtained by isolation (Fig. 5C and D, respectively). These data highlight that the combination of agar plating and dilution-to-extinction are necessary to capture a greater proportion of richness from the endogenous microbial community.

### Culture-based approaches yield isolates belonging to the key taxa found by culture-independent approaches

**Figure 6:**
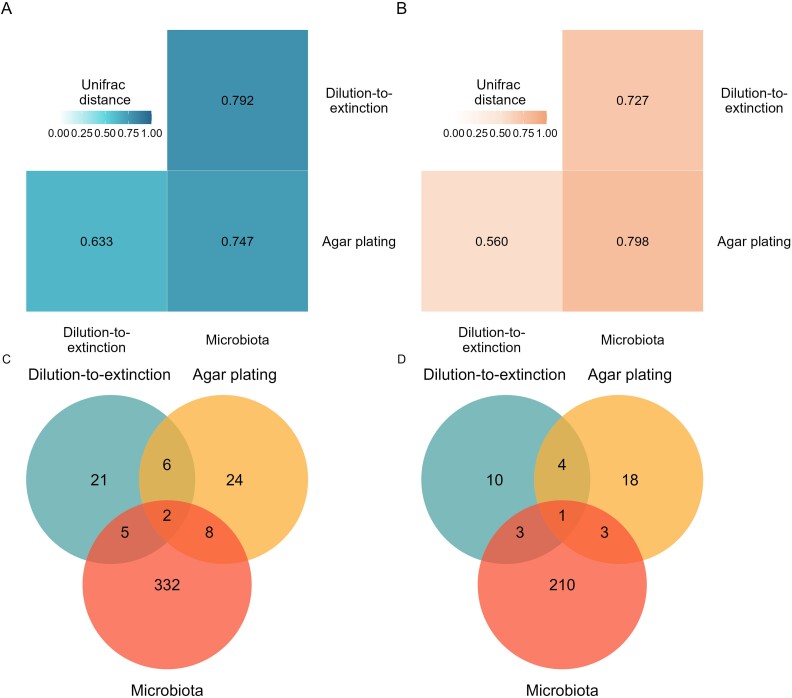
Comparison of oak microbial isolates obtained by agar plating and dilution-to-extinction and the endogenous oak microbial community analysed by a culture-independent approach. (A) Unifrac distance between bacterial isolates characterized by 16S rRNA gene profiling and the bacterial fraction of the endogenous oak microbiota, (B) Unifrac distance between fungal isolates characterized by ITS profiling and the fungal fraction of the endogenous oak microbiota, (C and D) number of bacterial and fungal ASVs, respectively, detected in oak microbial isolates obtained by agar plating and dilution-to-extinction and ASVs detected by culture-independent analysis of the oak microbiota.

We compared the cultivated fraction of the oak microbiota with the composition of the endogenous microbiota obtained by culture-independent analysis of the same tissue samples (single gene community profiling). Unifrac distances demonstrated that the profile of bacterial isolates obtained using dilution-to-extinction was more similar to the endogenous microbiota obtained by cultivation-independent analysis than the isolates profile obtained using agar plating. For fungi, agar plating generated isolates more similar to the endogenous fungal profile than dilution-to-extinction. However, the profiles of isolates captured by agar plating and dilution-to-extinction were more similar to each other than to the oak microbiota profile (Fig. 6A and B).

To investigate what component of the endogenous microbiota was cultured with the two isolation approaches, we compared the ASVs obtained by isolation with both the top 100 most abundant ASVs and the entire set of ASVs obtained by culture-independent analysis. The isolation methods only captured 15 bacterial ASVs (4%) and 7 fungal ASVs (3%) of the total ASVs in the culture-independent analysis (Fig. 6C and D). Interestingly, only 9 of these bacterial ASVs were in the top 100 most abundant ASVs (Supplementary Fig. 2), demonstrating that isolation approaches also yielded low abundant ASVs.

We also found that 57 bacterial ASVs and 32 fungal ASVs obtained by isolation methods were not detected by culture-independent analysis (Fig. 6C and D). To confirm that these ASVs still represented actual isolates rather than uncultured traces of microbial biomass from oak tissue samples, we analysed the proportion of reads and ASVs obtained from inoculated media where no microbial growth was observed (see Methods). These isolation negative controls yielded no sequences or ASVs (representing 0.2% reads of the bacterial data set and 0% reads of the fungal data set), suggesting that the ASVs captured through the isolation methods originated from genuine members of the oak microbiota and not from external biomass sources.

**Figure 7:**
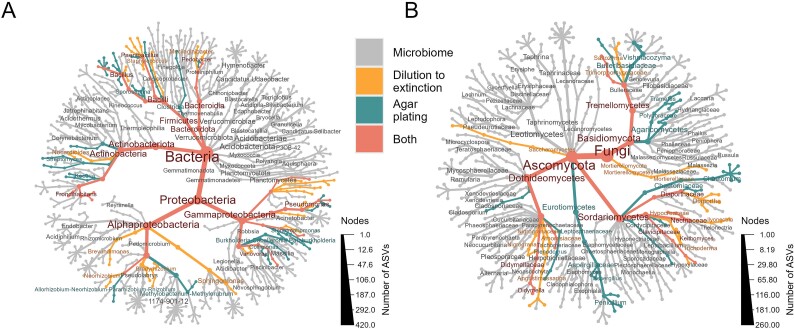
Taxonomic composition at phylum, class and genus levels of the members of the oak microbiota isolated by agar plating and dilution-to-extinction compared with cultured-independent analysis of the oak endogenous microbiota. (A) Bacterial and (B) fungal isolates obtained from oak tissue were analysed by 16S rRNA gene and ITS community profiling, respectively, and compared against the community profiles of the endogenous microbiota present in the same oak tissue samples. Taxa obtained by culture-dependent and culture-independent sequencing are depicted using the colour scales shown in the figure legend.

Despite the low overlap between ASVs from culture-dependent and culture-independent analysis, microbial isolation captured a broad range of taxonomic groups of the endogenous oak microbiota. For instance, the most abundant phyla in culture-independent analysis, represented as thicker nodes and edges in the heat taxa trees, were Proteobacteria, Actinobacteriota, and Firmicutes, all of which are represented by the bacterial isolates obtained in this study (Fig. 7A). At the genus level, we isolated members of bacterial genera that are commonly dominant in the plant microbiome, such as *Pseudomonas*, *Curtobacterium,* and *Frondihabitans* [63] or commonly found in oak trees such as *Burkholderia*, *Bacillus*, and *Streptomyces* ([33], 2017). Similarly, the most abundant fungal taxa in the oak microbiota determined by culture-independent analysis were the phyla Ascomycota and Basidiomycota, which were both captured by the isolation methods (Fig. 7B). At the genus level, we obtained representatives of *Chaetomium*, *Penicillium,* and *Trametes*, which are also commonly found in plant and soil microbiota. Consequently, at the genus- and phylum-level, culture-based and culture-independent approaches identified the same key taxa.

## Discussion

Isolating representative members of the microbiome remains critical to uncovering the true microbial diversity in multiple environments, studying ecological roles, and confirming metabolic and physiological functions of the microbiota [16]. Here, we tested the efficacy of two low-cost, easily implementable isolation methods to isolate bacteria and fungi from the oak microbiota. Our results demonstrate that the number and taxonomic richness of oak isolates are influenced by agar type, incubation conditions, and type of sample inoculated into the growth media. The use of media with varying nutrient compositions increases the likelihood of simultaneously fulfilling the diverse nutritional requirements of multiple groups of microorganisms and facilitates the isolation of slow-growing or rare microorganisms [13, 15, 70].

We also tested the dilution-to-extinction method for isolation of bacterial and fungal microbiota. Using this approach, bacterial, and fungal isolates were obtained from root/rhizosphere and leaf samples, but fewer stem isolates were obtained compared to these tissues. This could be attributed to: (i) difficulties in macerating woody inner bark tissue and recovering microorganisms attached to the wood material [71, 72], (ii) lower microbial cell density in the stem compared to other tissue types, (iii) stem microbiota requiring different growth requirements to the ones tested here for dilution-to-extinction, such as anaerobic conditions [73] and different agar formulations [74]. Bacterial growth, specifically, could have been limited due to essential interdependencies or syntrophic interactions in the stem microbiota [15]. Such interactions have been observed between specific strains in plant stem and in other plant compartments [75–78]. Fungal isolates were not obtained by dilution-to-extinction, potentially due to the reasons described above, and the high dilution factor used [37].

Despite the low number of stem isolates, the taxonomic richness obtained by dilution-to-extinction was higher than any individual agar medium used. In agar plating, cells are fixed onto a solid structure which can limit resources and the ability of cells to interact with distant neighbors, while in dilution-to-extinction, cells tend to have equal access to resources and interactions can occur more globally [79]. This allows capturing higher microbial richness, as liquid-based cultures facilitate random co-culturing of low diversity communities within a well, and can isolate more numerous microorganisms whose growth in monoculture is difficult to attain [14].

Overall, the composition of the isolates obtained by agar plating and dilution-to-extinction was significantly distinctive, with only 12% of the ASVs captured by both methods and some bacterial and fungal genera being particularly abundant in only one of the isolation methods. This suggests that each isolation method captured a different fraction of the oak microbiota and that the combination of the two had a positive effect in recovering greater taxonomic richness in the collection of isolates. Combinations of agar plating and dilution-to-extinction have been previously used to isolate plant microbiota [13, 20, 21, 80], but the effects of the isolation method on the overall number and richness of the cultivable tree microbiota have not been explored.

Despite the efficacy of a combination of isolation methods to recover high taxonomic richness, the proportion of ASVs captured by isolation with respect to the culture-independent analysis was lower than in previous reports [1, 20, 81]. Cross-referencing ASVs from different datasets obtained by amplicon sequencing can be influenced by multiple external factors such as primer biases, mismatches with commonly used primer sets that impede the detection of certain groups [15], number and variability of gene copies [82] and inherent biases related to PCR [63]. In addition, the isolation throughput in this study was kept at a limited scale (~360 bacterial and ~ 600 fungal colonies/wells) for the purpose of assessing isolation methods only. Studies reporting percentages of ASVs from isolates vs culture-independent analysis as high as 80% are based on a number of strains 20 times higher than in this study [1]. Therefore, it is not surprising that a small portion of the total ASVs overlapped between culture-independent analysis and isolation methods. Nonetheless, even with a low number of isolates, we were able to capture some ecological patterns of the endogenous microbiota, such as dominant groups in the plant and oak microbiota (i.e. bacterial phyla Proteobacteria, Actinobacteria, Acidobacteria, and Verrucomicrobia, and fungal phyla Basidiomycota and Ascomycota) [20, 83], and the distinctive taxonomic richness and composition of bacterial and fungal communities between plant compartments which tends to be higher in the root/rhizosphere than in the phyllosphere (leaf and stem) [84].

We also found that some microorganisms captured in the isolation methods were not present in the culture-independent analysis. Similar results have been reported in human gut microbiota studies comparing culture-dependent and culture-independent analysis [22, 81]. Some ASVs originally present in the endogenous microbiota can be rare or not populous enough to be detectable by culture-independent sequencing, however, they can potentially be enriched to detectable levels when coupling isolation methods with amplicon sequencing [2, 22, 81, 85]. This highlights the fact that isolation studies can complement and tackle some of the shortcomings of culture-independent microbiome studies [22, 86].

Here we demonstrate that agar plating and dilution-to-extinction-based isolation approaches can be optimized to capture different fractions of the endogenous oak microbiota. When used in combination, these approaches facilitated the recovery of an increased taxonomic richness of culturable microbiota. Large-scale microbial isolation studies coupled with “omics” data are increasingly being applied to drive advances in human microbiome research [1, 87], and in the rhizosphere and leaf tissues of model plant and crop species [13, 20, 21, 37]. In this study, we tested two contrasting isolation approaches (agar plating and dilution-to-extinction), different medium compositions, and incubation conditions, on three different tree tissue types (leaf, stem, root/rhizosphere), guided by culture-independent analysis of the microbial communities of the same tissue samples, to address key knowledge gaps on the composition and culturable fraction of tree-associated microbiota. The work highlights the advantages and disadvantages of different isolation approaches and demonstrates that the incorporation of contrasting isolation methodologies is a promising strategy to produce taxonomically richer microbial culture collections. Further research should validate these methods in other systems and focus on developing growth conditions to target specific taxa and cover a wider range of the members of the endogenous microbiota. Efforts to isolate members of the microbiota need to be increased as this will directly impact our knowledge of the microbiomes in a wide range of hosts and environments and our ability to progress research on the design and development of microbial transplants and microbiome engineering research.

## Supplementary Material

Supplementary_Fig1_ycaf019

Supplementary_Fig2_ycaf019

Supplementary_material_ycaf019

## Data Availability

Demultiplexed sequences are available in the European Nucleotide Archive (Project accession number PRJEB82441).
