# Supplementary figures and images for: Evaluating agar-plating and dilution-to-extinction isolation methods for generating oak-associated microbial culture collections
Source: ISME Commun. 2025 Feb 11;5(1):ycaf019. doi: 10.1093/ismeco/ycaf019 (PMC11878766; doi:10.1093/ismeco/ycaf019)

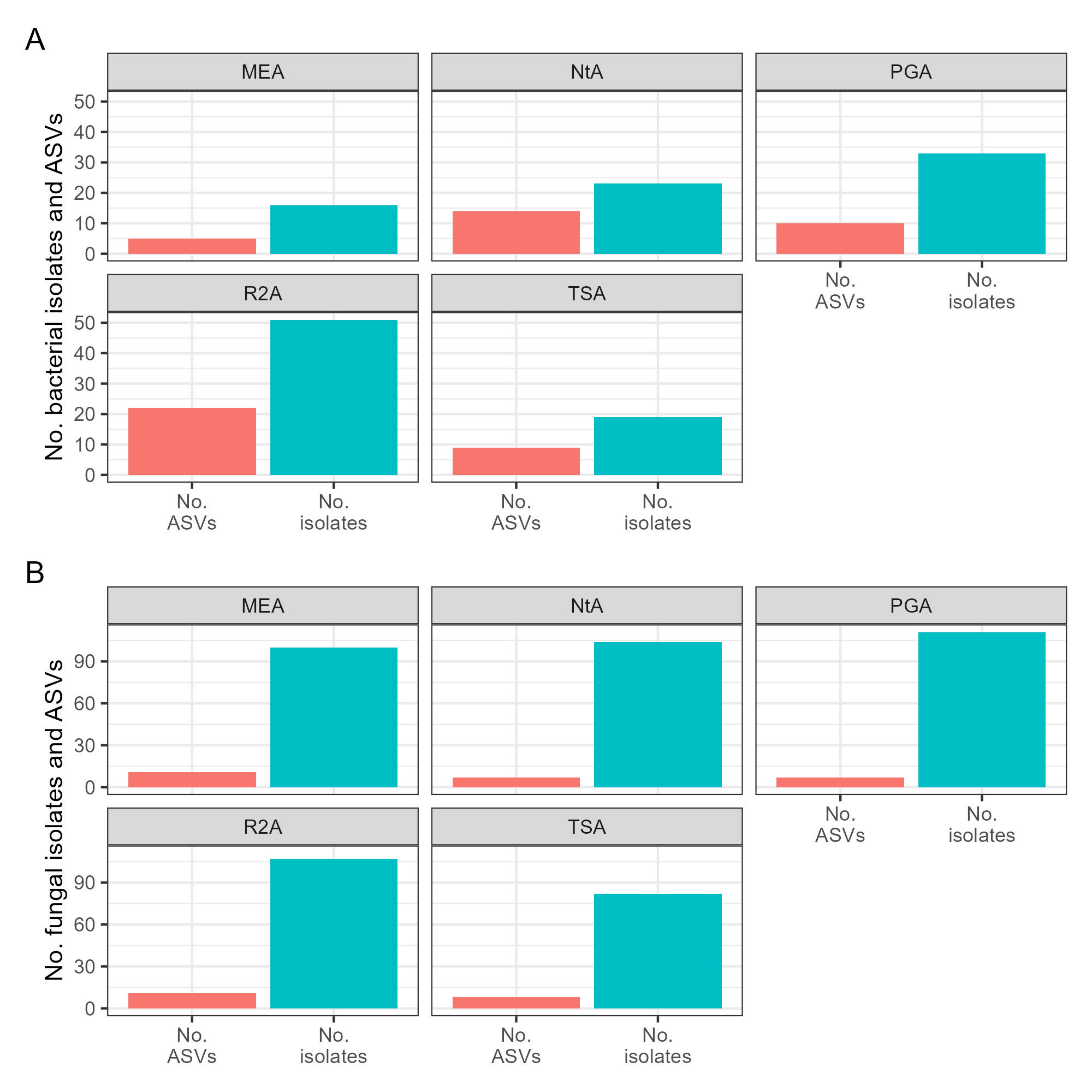

Supplement: Supplementary_Fig1_ycaf019 [file supplementary_fig1_ycaf019.jpeg]
