# Supplementary material for: Evaluating agar-plating and dilution-to-extinction isolation methods for generating oak-associated microbial culture collections
Source: ISME Commun. 2025 Feb 11;5(1):ycaf019. doi: 10.1093/ismeco/ycaf019 (PMC11878766; doi:10.1093/ismeco/ycaf019)

Supplementary Table 1. Pools of bacterial and fungal colonies isolated by agar plating and pools of wells of 96-well plates with microbial growth obtained by dilution-to-extinction. Bacterial and fungal isolates obtained in agar plating were pooled by isolation method, tissue type, growth medium and incubation condition/type of sample inoculated (cell suspensions or stem pieces). Wells with bacterial and fungal growth obtained by dilution-to-extinction was pooled per dilution. The growth media used were: nutrient agar (NtA), malt extract agar (MEA), Reasoner's 2 agar (R2A), potato-glucose agar (PGA), tryptone-soy agar (TSA), fastidious anaerobe media (FAA), 0.1X tryptone-soy broth (TSB) and 0.1X malt extract agar supplemented with streptomycin (MEA+S).

**Agar plating**						
**Sample**	**Tissue**	**Medium**	**Incubation**	**Sample preparation**	**Replicate**	**Isolation method**
1	Root/rhizosphere	NtA	Aerobic	Cell suspension diluted 10^-3 and 10^-4	a, b	Agar plating
2	Root/rhizosphere	TSA	Aerobic	Cell suspension diluted 10^-3 and 10^-4	a, b	Agar plating
3	Root/rhizosphere	MEA	Aerobic	Cell suspension diluted 10^-3 and 10^-4	a, b	Agar plating
4	Root/rhizosphere	PGA	Aerobic	Cell suspension diluted 10^-3 and 10^-4	a, b	Agar plating
5	Root/rhizosphere	R2A	Aerobic	Cell suspension diluted 10^-3 and 10^-4	a	Agar plating
6	Root/rhizosphere	R2A	Aerobic	Cell suspension diluted 10^-3 and 10^-4	b	Agar plating
7	Leaf	NtA	Aerobic	Dilutions -1 and -2	a, b	Agar plating
8	Leaf	TSA	Aerobic	Cell suspension diluted 10^-1 and 10^-2	a, b	Agar plating
9	Leaf	MEA	Aerobic	Cell suspension diluted 10^-1 and 10^-2	a, b	Agar plating
10	Leaf	PGA	Aerobic	Cell suspension diluted 10^-1 and 10^-2	a, b	Agar plating
11	Leaf	R2A	Aerobic	Cell suspension diluted 10^-1 and 10^-2	a	Agar plating
12	Leaf	R2A	Aerobic	Cell suspension diluted 10^-1 and 10^-2	b	Agar plating
13	Stem	NtA	Aerobic	Undiluted cell suspension	a, b	Agar plating
14	Stem	TSA	Aerobic	Undiluted cell suspension	a, b	Agar plating
15	Stem	MEA	Aerobic	Undiluted cell suspension	a, b	Agar plating
16	Stem	PGA	Aerobic	Undiluted cell suspension	a, b	Agar plating
17	Stem	R2A	Aerobic	Undiluted cell suspension	a	Agar plating
18	Stem	R2A	Aerobic	Undiluted cell suspension	b	Agar plating
19	Stem	NtA, TSA, MEA, PGA, R2A	Aerobic	Stem pieces		Agar plating targeting stem microbiota
20	Stem	NtA, TSA, MEA, PGA, R2A, FAA	Anaerobic	Undiluted cell suspension	a, b	Agar plating targeting stem microbiota

21	Stem	NtA, TSA, MEA, PGA, R2A, FAA	Anaerobic	Stem pieces		Agar plating targeting stem microbiota
**Dilution to extinction for bacteria**						
**Sample**	**Tissue**	**Medium**	**Incubation**	**Sample preparation**	**Replicate**	**Isolation method**
22	Root/rhizosphere	TSB	Aerobic	Cell suspension diluted from 10^-1 to 10^-4	1, 2	Dilution to extinction (Bacteria)
23	Root/rhizosphere	TSB	Aerobic	Cell suspension diluted to 10^-5	1	Dilution to extinction (Bacteria)
24	Root/rhizosphere	TSB	Aerobic	Cell suspension diluted to 10^-5	2	Dilution to extinction (Bacteria)
25	Leaf	TSB	Aerobic	Cell suspension diluted from 10^-1 to 10^-2	1, 2	Dilution to extinction (Bacteria)
26	Leaf	TSB	Aerobic	Cell suspension diluted to 10^-3	1	Dilution to extinction (Bacteria)
27	Leaf	TSB	Aerobic	Cell suspension diluted to 10^-3	2	Dilution to extinction (Bacteria)
29	Stem	TSB	Aerobic	Cell suspension diluted to 10^-1	1	Dilution to extinction (Bacteria)
30	Stem	TSB	Aerobic	Cell suspension diluted to 10^-1	2	Dilution to extinction (Bacteria)
**Dilution to extinction for fungi**						
**Sample**	**Tissue**	**Medium**	**Incubation**	**Sample preparation**	**Replicate**	**Isolation method**
31	Root/rhizosphere	MEA+S	Aerobic	Cell suspension diluted to 10^-1	1, 2	Dilution to extinction (Fungi)
32	Root/rhizosphere	MEA+S	Aerobic	Cell suspension diluted to 10^-2	1, 2	Dilution to extinction (Fungi)
33	Leaf	MEA+S	Aerobic	Cell suspension diluted to 10^-1	1, 2	Dilution to extinction (Fungi)
34	Leaf	MEA+S	Aerobic	Cell suspension diluted to 10^-2	1, 2	Dilution to extinction (Fungi)
**Controls**						
**Sample**	**Tissue**	**Medium**	**Incubation**	**Sample preparation**	**Replicate**	**Isolation method**
35	Root/rhizosphere	Any	Aerobic	Any agar plate with no growth		Agar plating - Almost no growth
36	Root/rhizosphere	TSA	Aerobic	Wells of dilution 10^-5 with no growth	1	Dilution to extinction - Inoculated microwells with no growth
37	Leaf	Any	Aerobic	Any agar plate with no growth		Agar plating - Almost no growth
38	Leaf	TSA	Aerobic	Wells of dilution 10^-3 with no growth	1, 2	Dilution to extinction - Inoculated microwells with no growth
39	Stem	Any	Aerobic	Any agar plate with no growth		Agar plating - Almost no growth

Supplementary Table 2. Total number of reads in the sequencing data set at each curation step after the sample demultiplexing and analysis in the nf-core/ampliseq pipeline.

Curation steps	Number of reads after each step	
	16s rRNA gene	ITS
Total number of reads	261,176	278,009
decontam package	251,441	270,586
Mitochondria reads removal	185,239	270,586
Cyanobacteria/chloroplast reads removal	184,156	270,586
Removal of reads in DNA/PCR negative controls	181,729	268,014
Removal of ASVs with less than 100 reads	171,861	260,221

Table 3. Comparison number of isolates and ASVs between growth medium (NtA, R2A, PGA, TSA, MEA) and tissue types (root/rhizosphere, leaf, stem). Statistical differences were tested using a non-parametric Kruskall-Wallis test, followed by Dunn's test between significantly different groups.

Groups compared	Chi-squared	Degrees of freedom	p-value	Test	Organism
Bacterial isolates vs medium	5.51	4	0.24	Kruskal-Wallis	Bacteria
Bacterial ASVs vs medium	2.12	4	0.71	Kruskal-Wallis	Bacteria
**Bacterial isolates vs tissue**	17.74	2	**0.00**	Kruskal-Wallis	Bacteria
**Bacterial ASVs vs tissue**	7.70	2	**0.02**	Kruskal-Wallis	Bacteria
Fungal isolates vs medium	3.26	4	0.51	Kruskal-Wallis	Fungi
Fungal ASVs vs medium	4.50	4	0.34	Kruskal-Wallis	Fungi
**Fungal isolates vs tissue**	19.87	2	**0.00**	Kruskal-Wallis	Fungi
Fungal ASVs vs tissue	3.97	2	0.14	Kruskal-Wallis	Fungi
**Pairwise comparison between tissue types**	**Z**	**p-unadj**	**p-adj**	**Variable**	**Organism**
Leaf – Root/Rhizosphere	-1.09	0.28	0.28	Number of isolates	Bacteria
**Leaf – Stem**	**2.98**	**0.00**	**0.00**	Number of isolates	Bacteria
**Root/Rhizosphere – Stem**	**4.07**	**0.00**	**0.00**	Number of isolates	Bacteria
Leaf – Root/Rhizosphere	-1.92	0.05	0.08	Number of ASVs	Fungi
Leaf – stem	0.77	0.44	0.44	Number of ASVs	Fungi
Root/Rhizosphere – stem	2.69	0.01	0.02	Number of ASVs	Fungi
**Leaf - Root/Rhizosphere**	**3.56**	**0.00**	**0.00**	Number of isolates	Fungi
**Leaf – Stem**	**4.10**	**0.00**	**0.00**	Number of isolates	Fungi
Root/Rhizosphere – Stem	0.54	0.59	0.59	Number of isolates	Fungi

Supplementary table 4. Permanova analysis of bacterial and fungal isolates obtained from oak root/rhizosphere, stem and leaf samples using agar plating with different growth media. Bacterial and fungal isolates were characterized by single-gene profiling using the 16s rRNA gene and ITS, respectively.

Group	Df	Sum of squares	R2	F	Pr(>F)	Organism
Medium	4	1.53	0.22	1.27	0.16	Bacteria
Tissue	2	1.83	0.26	3.05	**0.00**	Bacteria
Medium:tissue	7	2.74	0.39	1.30	0.13	Bacteria
Residual	3	0.90	0.13	NA	NA	Bacteria
Total	16	7.01	1.00	NA	NA	Bacteria
Medium	4	1.54	0.21	1.36	0.10	Fungi
Tissue	2	2.27	0.30	4.02	**0.00**	Fungi
Medium:tissue	8	2.86	0.38	1.27	0.13	Fungi
Residual	3	0.85	0.11	NA	NA	Fungi
Total	17	7.52	1.00	NA	NA	Fungi

Supplementary Table 5. Bacterial and fungal genera represented in the oak microbial isolates obtained in a single type of medium. Microbial isolates from oak root/rhizosphere, stem and leaf tissue were isolated in five types of growth medium. Bacterial and fungal isolates were harvested and characterized by 16S rRNA gene and ITS profiling, respectively, and the genera represented in the set of isolates grown in only one type of growth medium was determined. The media used were nutrient agar (NtA), malt extract agar (MEA), Reasoner's 2 agar (R2A), potato-glucose agar (PGA), tryptone-soy agar (TSA).

Genus	Medium	Tissue	Organism
Burkholderia-Caballeronia-Paraburkholderia	MEA	Root/Rhizosphere	Bacteria
Methylobacterium-Methylorubrum	PGA	Stem	Bacteria
Kocuria	R2A	Stem	Bacteria
Kitasatospora	PGA	Root/Rhizosphere	Bacteria
Streptomyces	PGA	Root/Rhizosphere	Bacteria
Collimonas	PGA	Root/Rhizosphere	Bacteria
Paenibacillus	TSA	Root/Rhizosphere	Bacteria
Allorhizobium-Neorhizobium-ParарhizobiumRhizobium	R2A	Root/Rhizosphere	Bacteria
Luteibacter	R2A	Root/Rhizosphere	Bacteria
Serratia	R2A	Root/Rhizosphere	Bacteria
Unassigned	R2A	Root/Rhizosphere	Bacteria
Pedobacter	R2A	Root/Rhizosphere	Bacteria
Rhodococcus	R2A	Root/Rhizosphere	Bacteria
Dyella	R2A	Root/Rhizosphere	Bacteria
Clonostachys	NtA	Root/Rhizosphere	Fungi
Aspergillus	R2A	Stem	Fungi
Papiliotrema	PGA	Leaf	Fungi
Vishniacozyma	R2A	Leaf	Fungi
Plenodomus	R2A	Leaf	Fungi
Crustomyces	MEA	Root/Rhizosphere	Fungi
Keithomyces	MEA	Root/Rhizosphere	Fungi
Libertasomyces	NtA	Leaf	Fungi

Supplementary Table 6. Kruskal-Wallis test to compare the number of oak microbial isolates obtained in diluted and undiluted growth medium and supplemented and non-supplemented growth medium. Oak leaf extract to a final concentration of 2% was used. The number of bacterial and fungal colonies obtained in each condition was tested using the non-parametric Kruskal-Wallis test.

Variable compared	Chi-squared	Degrees of freedom	p-value	Test
Bacterial isolates vs Supplement	0.39	1	0.53	Kruskal-Wallis
**Bacterial isolates vs Dilution**	**9.73**	**1**	**0.00**	Kruskal-Wallis
Fungal isolates vs Supplement	0.85	1	0.36	Kruskal-Wallis
Fungal isolates vs Dilution	0.71	1	0.40	Kruskal-Wallis

Supplementary Table 7. Kruskal-Wallis test comparing the number of bacterial and fungal isolates and ASVs obtained by agar plating under different growth conditions targeting stem microbiota. Bacterial isolates were obtained in (i) a combination of five growth media (NtA, MEA, PGA, R2A and TSA) under aerobic incubation and injection of stem cell suspensions and (ii) a combination of six growth media (addition of FAA) under anaerobic conditions, and inoculation of stem cell suspensions and stem pieces (stem targeting conditions). Bacterial and fungal ASVs were determined by single-gene profiling using the 16S rRNA gene, for characterization of bacterial isolates, and ITS, for characterization of fungal isolates.

Variable compared	Chi-squared	Degrees of freedom	p-value	Test
Bacterial isolates in different stem growth conditions	0.75	1	0.39	Kruskal-Wallis
Bacterial ASVs in different stem growth conditions	2.89	1	0.09	Kruskal-Wallis
Fungal isolates in different stem growth conditions	6.13	1	**0.01**	Kruskal-Wallis
Fungal ASVs in different stem growth conditions	1.65	1	0.20	Kruskal-Wallis

Supplementary Table 8. Percentage of wells displaying bacterial and fungal growth in a dilution-to-extinction approach to cultivate microbial isolation from oak-associated microbiota. Cell suspensions prepared from root/rhizosphere, leaf and stem tissue samples were diluted in growth medium and each dilution was dispensed in 24 wells of a 96-well plate. After 1 week under aerobic incubation, the number of wells inoculated which displayed microbial growth was registered. The growth media used was tryptone soy broth, for bacterial isolation and malt extract agar supplemented with streptomycin (50 mg/uL final concentration), for fungal isolation.

Dilution factor	% Wells with bacterial growth			% Wells with fungal growth		
	Root/Rhizosphere	Leaf	Stem	Root/Rhizosphere	Leaf	Stem
10^-1	100%	100%	100%	83%	96%	0%
10^-2	100%	100%	4%	50%	46%	0%
10^-3	100%	88%	0%	17%	8%	0%
10^-4	92%	8%	0%	0%	0%	0%
10^-5	46%	4%	0%	0%	0%	0%
10^-6	0%	0%	0%	0%	0%	0%
10^-7	0%	0%	0%	0%	0%	0%
10^-8	0%	0%	0%	0%	0%	0%

Supplementary Table 9. Permanova analysis of bacterial and fungal isolates obtained by dilution-to-extinction from oak root/rhizosphere, stem and leaf microbiota tissue samples. Bacterial and fungal isolates were characterized by single-gene profiling using the 16s rRNA gene and ITS, respectively.

Parameter	Degrees of freedom	Sum Of Squares	R2	F	Pr(>F)	Organism
**Tissue**	**2**	**1.21**	**0.43**	**1.49**	**0.01**	**Bacteria**
Residual	4	1.62	0.57	NA	NA	Bacteria
Total	6	2.83	1.00	NA	NA	Bacteria
Tissue	1	0.67	0.50	1.99	0.33	Fungi
Residual	2	0.67	0.50	NA	NA	Fungi
Total	3	1.33	1.00	NA	NA	Fungi

Supplementary Table 10. Kruskal-Wallis test comparing the number of bacterial and fungal ASVs obtained from the oak microbiota by dilution-to-extinction with isolates obtained in different types of agar media. Microbial isolates from oak-associated microbiota (root/rhizosphere, stem and leaf) obtained by dilution-to-extinction and by agar plating in five different agar media were characterized through single-gene profiling using the 16S rRNA, for bacteria, and the ITS, for fungi. The number of ASVs obtained by dilution-to-extinction and each of the agar media was tested. Pairwise comparisons were tested using a post-hoc Dunn's test to compare significantly distinct groups. The agar media used for fungal dilution-to-extinction was malt extract agar supplemented with streptomycin (50 mg/uL final concentration, MEA+S) and the five agar media used in agar plating were malt extract agar (MEA), Reasoner's 2 agar (R2A), potato glucose agar (PGA), nutrient agar (NtA) and tryptone soy agar (TSA).

Variable compared	Chi-squared	Degrees of freedom	p-value	Test	Organism
Number of bacterial ASV per medium	2.58	5.00	0.76	Kruskal-Wallis	Bacteria
**Number of fungal ASVs per medium**	**11.21**	**5.00**	**0.05**	**Kruskal-Wallis**	**Fungi**
**Pairwise comparison**	**Z**	**p-unadj**	**p-adj**	**Variable**	**Organism**
MEA - MEA+S	-0.95	0.34	0.64	Number of fungal ASVs	Fungi
MEA - Nta	1.28	0.20	0.50	Number of fungal ASVs	Fungi
MEA+S – NtA	2.32	0.02	0.15	Number of fungal ASVs	Fungi
MEA – PGA	1.03	0.31	0.65	Number of fungal ASVs	Fungi
MEA+S – PGA	2.05	0.04	0.20	Number of fungal ASVs	Fungi
NtA – PGA	-0.26	0.80	0.85	Number of fungal ASVs	Fungi
MEA - R2A	1.74	0.08	0.25	Number of fungal ASVs	Fungi
**MEA+S - R2A**	3.03	0.00	**0.04**	Number of fungal ASVs	Fungi
NtA - R2A	0.26	0.80	0.92	Number of fungal ASVs	Fungi
PGA - R2A	0.55	0.58	0.79	Number of fungal ASVs	Fungi
MEA – TSA	0.80	0.42	0.63	Number of fungal ASVs	Fungi
MEA+S – TSA	1.81	0.07	0.27	Number of fungal ASVs	Fungi
NtA – TSA	-0.48	0.63	0.79	Number of fungal ASVs	Fungi
PGA – TSA	-0.22	0.82	0.82	Number of fungal ASVs	Fungi
R2A – TSA	-0.81	0.42	0.69	Number of fungal ASVs	Fungi

Supplementary Table 11. Permanova analysis of bacterial and fungal isolates obtained by agar plating and dilution-to-extinction from oak root/rhizosphere, stem and leaf tissue samples. Bacterial and fungal isolates were characterized by single-gene profiling using the 16s rRNA gene and ITS, respectively.

Parameter	Degrees of freedom	Sum of Squares	R2	F	Pr.F	Organism
**Isolation method**	1	0.60	0.05	1.59	**0.04**	Bacteria
**Tissue**	2	2.23	0.19	2.98	**0.00**	Bacteria
**Isolation method:Tissue**	2	1.11	0.09	1.48	**0.02**	Bacteria
Residual	21	7.89	0.67	NA	NA	Bacteria
Total	26	11.83	1.00	NA	NA	Bacteria
**Isolation method**	1	0.77	0.07	2.45	**0.00**	Fungi
**Tissue**	2	2.96	0.28	4.70	**0.00**	Fungi
**Isolation_method:Tissue**	1	0.63	0.06	2.01	**0.02**	Fungi
Residual	2	6.29	0.59	NA	NA	Fungi
Total	2	10.65	1.00	NA	NA	Fungi

Supplementary Table 12. Fisher's exact test comparing differences in the number of ASVs per genus, obtained by agar plating and dilution-to-extinction from oak tissue samples. Microbial isolation from oak root/rhizosphere, stem and leaf samples was conducted by agar plating and dilution-to-extinction. Bacterial and fungal isolates obtained using the two methods were characterized by single-gene profiling using the 16S rRNA gene and ITS, respectively. Genus names significantly different between isolation methods are highlighted with an asterisk symbol.

Organism	Genus	p-value	Lower confidence interval	Upper confidence interval	Estimate	Null value
Bacteria	***Bacillus**	**0.04**	1.08	84.58	8.05	1
Bacteria	*Paenibacillus*	0.21	0.00	2.20	0.09	1
Bacteria	*Bradyrhizobium*	0.33	0.00	4.56	0.00	1
Bacteria	*Burkholderia-Caballeronia-Paraburkholderia*	0.33	0.22	Inf	Inf	1
Bacteria	*Sporosarcina*	0.33	0.22	Inf	Inf	1
Bacteria	*Pseudomonas*	0.62	0.26	32.60	2.60	1
Bacteria	*AAP99*	1.00	0.00	39.00	0.00	1
Bacteria	*Allorhizobium-Neorhizobium-Pararhizobium-Rhizobium*	1.00	0.03	Inf	Inf	1
Bacteria	*Brevundimonas*	1.00	0.00	39.00	0.00	1
Bacteria	*Buttiauxella*	1.00	0.00	39.00	0.00	1
Bacteria	*Caldalkalibacillus*	1.00	0.03	Inf	Inf	1
Bacteria	*Clostridium* sensu stricto 1	1.00	0.03	Inf	Inf	1
Bacteria	*Collimonas*	1.00	0.03	Inf	Inf	1
Bacteria	*Curtobacterium*	1.00	0.01	155.93	1.00	1
Bacteria	*Dyella*	1.00	0.01	155.93	1.00	1
Bacteria	*Frateuria*	1.00	0.00	39.00	0.00	1
Bacteria	*Frondihabitans*	1.00	0.03	30.58	1.00	1
Bacteria	*Halotalea*	1.00	0.00	39.00	0.00	1
Bacteria	*Kitasatospora*	1.00	0.03	Inf	Inf	1
Bacteria	*Kocuria*	1.00	0.03	Inf	Inf	1
Bacteria	*Luteibacter*	1.00	0.03	Inf	Inf	1
Bacteria	*Methylobacterium-Methylorubrum*	1.00	0.03	Inf	Inf	1
Bacteria	*Micrococcus*	1.00	0.03	Inf	Inf	1
Bacteria	*Mucilaginibacter*	1.00	0.00	39.00	0.00	1
Bacteria	*Neorhizobium*	1.00	0.00	39.00	0.00	1
Bacteria	*Nocardioides*	1.00	0.00	39.00	0.00	1
Bacteria	*Pantoea*	1.00	0.00	39.00	0.00	1
Bacteria	*Pedobacter*	1.00	0.01	155.93	1.00	1
Bacteria	*Rhodococcus*	1.00	0.03	Inf	Inf	1
Bacteria	*Rhodopseudomonas*	1.00	0.00	39.00	0.00	1
Bacteria	*Serratia*	1.00	0.01	155.93	1.00	1

Bacteria	*Sphingomonas*	1.00	0.00	39.00	0.00	1
Bacteria	*Staphylococcus*	1.00	0.00	39.00	0.00	1
Bacteria	*Stenotrophomonas*	1.00	0.03	Inf	Inf	1
Bacteria	*Streptomyces*	1.00	0.03	Inf	Inf	1
Bacteria	*Unassigned*	1.00	0.00	14.84	0.32	1
Bacteria	*Variovorax*	1.00	0.07	350.56	3.11	1
Bacteria	*Yersinia*	1.00	0.00	39.00	0.00	1
**Fungi**	***Chaetomium**	**0.00**	3.52	Inf	Inf	1
Fungi	*Diaporthe*	0.10	0.00	1.54	0.00	1
Fungi	*Penicillium*	0.10	0.65	Inf	Inf	1
Fungi	*Trametes*	0.10	0.65	Inf	Inf	1
Fungi	*Angustimassarina*	0.33	0.00	4.56	0.00	1
Fungi	*Aspergillus*	1.00	0.03	Inf	Inf	1
Fungi	*Aureobasidium*	1.00	0.03	Inf	Inf	1
Fungi	*Clonostachys*	1.00	0.03	Inf	Inf	1
Fungi	*Crustomyces*	1.00	0.03	Inf	Inf	1
Fungi	*Didymella*	1.00	0.01	155.93	1.00	1
Fungi	*Ilyonectria*	1.00	0.00	39.00	0.00	1
Fungi	*Keithomyces*	1.00	0.00	14.84	0.32	1
Fungi	*Libertasomyces*	1.00	0.01	155.93	1.00	1
Fungi	*Nectria*	1.00	0.01	155.93	1.00	1
Fungi	*Nigrograna*	1.00	0.00	39.00	0.00	1
Fungi	*Ochrocladosporium*	1.00	0.00	39.00	0.00	1
Fungi	*Papiliotrema*	1.00	0.03	Inf	Inf	1
Fungi	*Phomopsis*	1.00	0.03	Inf	Inf	1
Fungi	*Plenodomus*	1.00	0.03	Inf	Inf	1
Fungi	*Podila*	1.00	0.00	39.00	0.00	1
Fungi	*Saitozyma*	1.00	0.00	39.00	0.00	1
Fungi	*Trichoderma*	1.00	0.00	39.00	0.00	1
Fungi	*Unassigned*	1.00	0.03	30.58	1.00	1
Fungi	*Vishniacozyma*	1.00	0.03	Inf	Inf	1

Supplementary Figure 1. Number of isolates and ASVs from the oak microbiota obtained by agar plating in different growth medium. A) Number of bacterial isolates and ASVs, B) Number of fungal isolates and ASVs. The number of bacterial and fungal ASVs representing the isolates obtained by agar plating in different growth medium was determined by 16S rRNA gene and ITS profiling. The growth media used were nutrient agar (NtA), malt extract agar (MEA), Reasoner's 2 agar (R2A), potato-glucose agar (PGA), tryptone-soy agar (TSA).

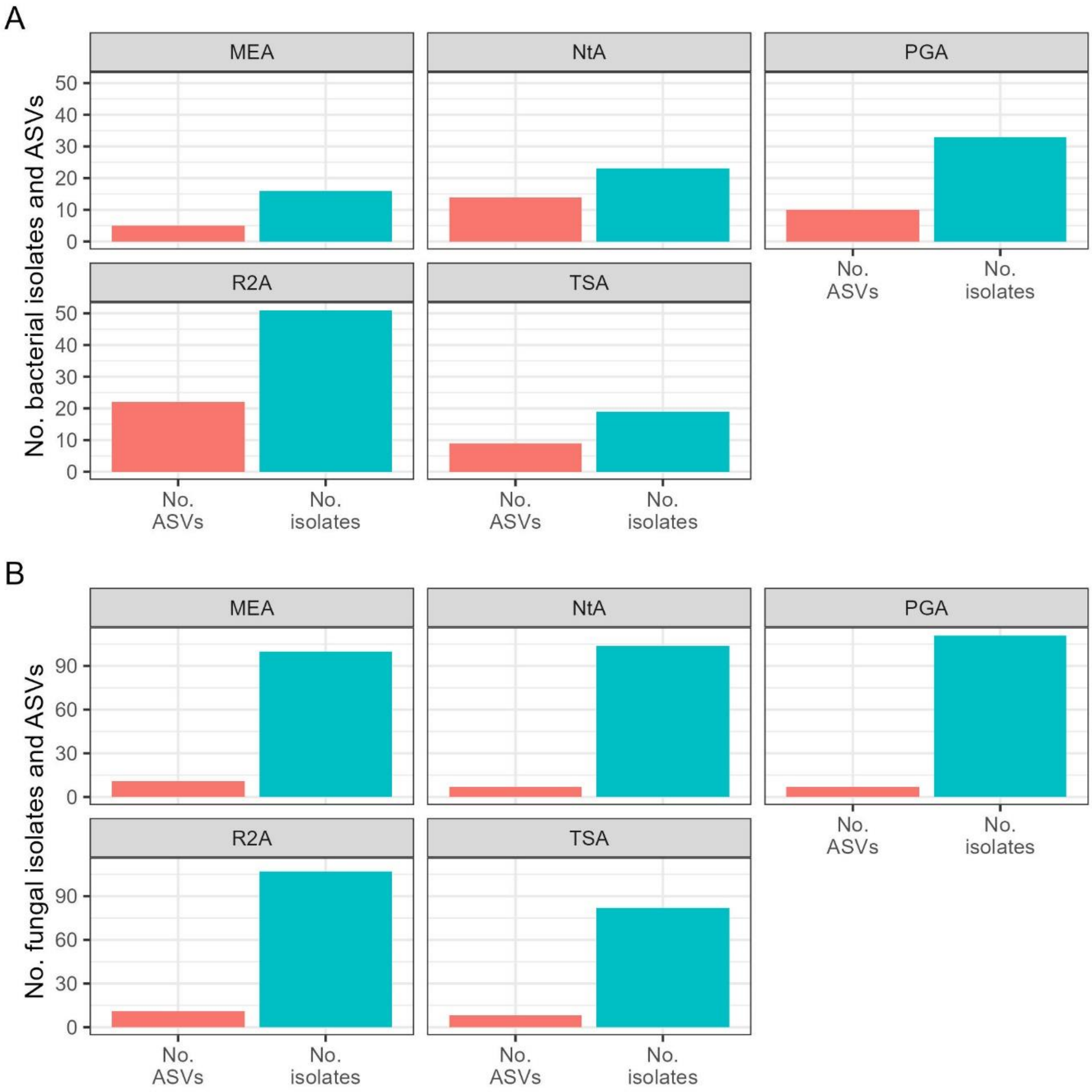

Supplementary Figure 2. Number of bacterial (A) and fungal (B) ASVs detected in oak microbial isolates versus the top 100 most abundant ASVs detected by culture-independent analysis of the oak microbiota. Bacterial and fungal isolates from oak tissue samples were obtained by dilution-to-extinction and agar plating. Pools of bacterial and fungal isolates were then characterized by 16S rRNA and ITS profiling, respectively, and the number of ASVs common to the most abundant ASVs in the culture-independent analysis of the same oak tissue samples was estimated.

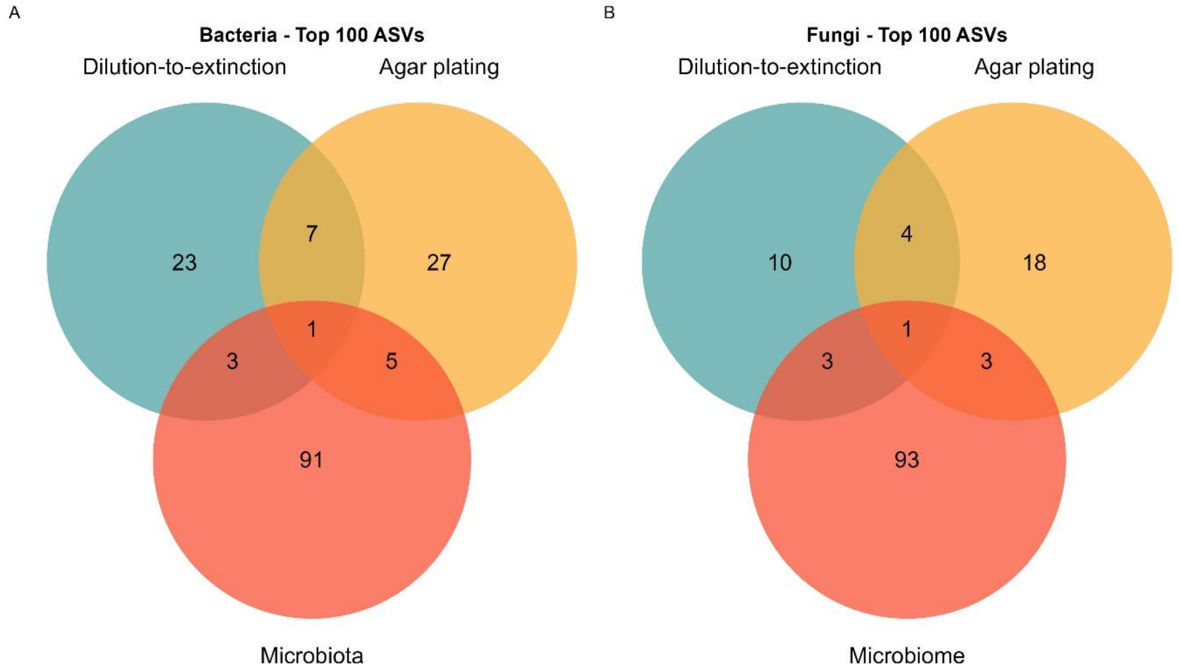

Supplement: Supplementary_material_ycaf019 [file supplementary_material_ycaf019.pdf]
